# Advancements in Digital Cytopathology Since COVID-19: Insights from a Narrative Review of Review Articles

**DOI:** 10.3390/healthcare13060657

**Published:** 2025-03-17

**Authors:** Daniele Giansanti

**Affiliations:** Centro TISP, ISS Via Regina Elena 299, 00161 Rome, Italy; daniele.giansanti@iss.it

**Keywords:** digital cytopathology, digitalization, AI, artificial intelligence

## Abstract

**Background/Objectives**: The integration of digitalization in cytopathology is an emerging field with transformative potential, aiming to enhance diagnostic precision and operational efficiency. This narrative review of reviews (NRR) seeks to identify prevailing themes, opportunities, challenges, and recommendations related to the process of digitalization in cytopathology. **Methods**: Utilizing a standardized checklist and quality control procedures, this review examines recent advancements and future implications in this domain. Twenty-one review studies were selected through a systematic process. **Results**: The results highlight key emerging trends, themes, opportunities, challenges, and recommendations in digital cytopathology. First, the study identifies pivotal themes that reflect the ongoing technological transformation, guiding future focus areas in the field. A major trend is the integration of artificial intelligence (AI), which is increasingly critical in improving diagnostic accuracy, streamlining workflows, and assisting decision making. Notably, emerging AI technologies like large language models (LLMs) and chatbots are expected to provide real-time support and automate tasks, though concerns around ethics and privacy must be addressed. The reviews also emphasize the need for standardized protocols, comprehensive training, and rigorous validation to ensure AI tools are reliable and effective across clinical settings. Lastly, digital cytopathology holds significant potential to improve healthcare accessibility, especially in remote areas, by enabling faster, more efficient diagnoses and fostering global collaboration through telepathology. **Conclusions**: Overall, this study highlights the transformative impact of digitalization in cytopathology, improving diagnostic accuracy, efficiency, and global accessibility through tools like whole-slide imaging and telepathology. While artificial intelligence plays a significant role, the broader focus is on integrating digital solutions to enhance workflows and collaboration. Addressing challenges such as standardization, training, and ethical considerations is crucial to fully realize the potential of these advancements.

## 1. Introduction

### 1.1. Evolution of Digital Pathology

The concept of digital cytology/cytopathology and digital histology/histopathology, both integral components of digital pathology, originated from telepathology in the late 20th century [1]. Telepathology initially relied on static image transmission [2,3] but evolved into a more dynamic field with robotic telepathology systems [4]. These systems allowed pathologists to remotely control microscopes, enabling real-time slide examination and remote diagnoses, particularly benefiting underserved regions [5,6].

Early telepathology faced challenges due to limited internet speeds, causing latency issues in robotic systems [7]. Problems such as synchronization errors in microscope functions (focus, zoom, and translation) led to inefficiencies. To address these issues, virtual microscopy was introduced as a deferred-time solution, enabling slide scanning, storage, and remote digital access [1].

### 1.2. Virtual Microscopy and Its Impact

Virtual microscopy revolutionized digital pathology by allowing high-resolution digital slide storage and remote analysis. The process begins with scanning physical slides using advanced digital scanners [8,9,10], which capture multi-layered images at various focal planes [11]. These images are then stitched together into a seamless digital replica, typically stored in formats like TIFF or JPEG2000 to preserve quality. Pathologists can analyze these slides on computer screens, adjusting focus, zoom, brightness, and contrast for detailed inspection.

A major advantage of virtual microscopy is its support for remote access and collaboration [12]. Once digitized, slides can be accessed via secure networks, allowing real-time consultations across geographical locations. This enhances efficiency in underserved areas, promotes international collaboration, and facilitates second opinions. Additionally, digital slides reduce the burden of physical storage and improve data retrieval for research, education, and clinical use [11,13,14]. However, successful implementation depends on infrastructure, training, and seamless integration into existing workflows [1,12].

### 1.3. Challenges in Implementation

Despite its potential, digital pathology integration has encountered challenges. A 2022 study revealed that digital slide scanning functioned as an additional step rather than replacing traditional workflows, increasing workload and operational costs [15]. Instead of streamlining processes, laboratories had to extend working hours and hire additional personnel for scanning procedures, contradicting initial cost-saving expectations.

Additionally, many institutions still use digital slides alongside traditional microscopy during the transition phase. This increases workload and financial burdens due to the need for specialized equipment, software, and staff training. Infrastructure investments, including high-resolution scanners and data storage, impose significant costs, making large-scale adoption difficult for smaller institutions [1,16,17,18].

Some evaluations, such as one conducted in Denmark, have found no significant cost reductions following digital pathology implementation. Instead, laboratory staff working hours increased due to the added time required for digital scanning and image analysis [16]. These findings highlight the need for a thorough cost–benefit analysis before large-scale adoption, as financial benefits may take time to materialize and vary between institutions.

### 1.4. Differences Between Digital Cytology and Histopathology

Digital cytology presents distinct challenges compared to digital histopathology due to differences in sample characteristics and imaging requirements. Cytology slides contain heterogeneous cell arrangements that demand sophisticated image processing. In histology, Z-stacking—capturing multiple focal layers to create a 3D image—is well-established [1,17]. However, in cytology, Z-stacking requires greater precision, making imaging more complex and data-intensive [1,17,18].

High-resolution Z-stack images consume significant storage space, more so than in fields like radiology [1,18]. Boschetto et al. [19] proposed software-based focus emulation to enhance image clarity, but its clinical implementation remains limited due to technical and logistical challenges.

AI applications in digital cytology are still developing, facing difficulties due to sample variability. While AI has advanced in histopathology, cytology’s complexity hampers standardization [18]. According to Giovagnoli and Giansanti [18], AI systems primarily designed for histology struggle with cytology’s diverse sample types, delaying widespread adoption.

Moreover, whole-slide imaging (WSI) [20] adoption in cytology remains limited. A Delphi expert consensus [21] and studies on thyroid cytopathology acknowledge WSI’s potential but note its slower clinical adoption in cytology. Chantziantoniou et al. [22] identified pathologist skepticism and the need for robust image analysis algorithms as barriers to digital cytopathology integration.

### 1.5. Defining Digital Cytology and Digital Cytopathology

Digital cytology and digital cytopathology, while related, have distinct roles. Digital cytology involves digitizing cell samples for remote viewing, analysis, and sharing, improving accessibility and workflow efficiency [23]. Digital cytopathology extends this by integrating advanced algorithms and AI to enhance disease diagnosis at the cellular level [24,25]. A recent study [26] defines digital cytology as a broader concept, encompassing not only digital slide review by cytopathologists but also workflow integration. This includes linking cytological cases with histopathology, supporting pathology–radiology conferences, and archiving cases for future research.

### 1.6. Growing Interest in Digital Cytopathology

A bibliometric analysis of PubMed using the terms (Cytopathology[Title/Abstract]) OR (Cytology[Title/Abstract]) AND (Digital[Title/Abstract]) identified 737 studies on digital cytology/cytopathology, with 41.9% (309 studies) published in the last five years [27]. This surge reflects growing interest in digital cytology, particularly post-COVID-19.

The pandemic accelerated digital pathology adoption, driving interest in virtual microscopy, remote consultation, and digital image sharing. The necessity for remote diagnostics fueled research and technological advancements, justifying the need for systematic reviews of recent trends, challenges, and innovations.

The purpose of this study is, therefore, to develop a narrative review of reviews (NRR) to analyze the current state of digitalization integration in cytopathology, with the following specific objectives:

- Analyze the overall bibliometric trends in digital cytopathology:
  The study aims to provide a comprehensive bibliometric analysis of research output in the field of digital cytopathology, focusing on trends and developments over time.
- Identify established themes and categories:
  Identify key areas of focus in recent reviews, such as AI applications, digital imaging, and automation in diagnostic processes.
- Examine opportunities and areas needing further improvements:
  Explore the potential benefits and areas needing improvement for integrating digital technologies into cytopathology, including advancements in diagnostic accuracy and workflow efficiency, as well as barriers like infrastructure and training.

## 2. Methods

### 2.1. Methodology Overview

The methodology comprises two main approaches: a narrative review of reviews and a bibliometric analysis. The narrative review synthesizes existing literature on digital cytopathology, focusing on advancements, challenges, and the role of artificial intelligence (AI). The bibliometric analysis examines historical research trends in digital cytopathology, particularly over the last decade (2014 to the present), with an emphasis on AI-related studies.

### 2.2. Narrative Review Selection and Qualification Process

The narrative review followed a structured selection process based on a standardized checklist for evaluating review articles [28]. Targeted searches were conducted using PubMed and Scopus with predefined composite search keys.

A qualification methodology was employed, using quality parameters described in [29] to determine study inclusion (Algorithm 1).
**Algorithm 1:** Selection Process for the NRR**Define search query:**○“((Cytopathology[Title/Abstract]) OR (Cytology[Title/Abstract]) AND (Digital*[Title/Abstract]))”**Conduct searches** in PubMed and Scopus using the defined query.**Select relevant studies** from peer-reviewed journals that focus on cytopathology. Priority was given to recent reviews that assess prior studies and reviews providing broad analyses integrating findings from previous works.**Evaluate each study** based on the following parameters:
○**N1:** Clear rationale in the introduction.○**N2:** Appropriate research design.○**N3:** Clearly described methodology.○**N4:** Well-presented results.○**N5:** Conclusions justified by results.○**N6:** Disclosure of conflicts of interest.
**Assign scores** to parameters **N1–N5** (scale of 1–5).**Assess N6** using a binary **Yes/No** measure.**Preselect studies** meeting the following criteria:
○**N6 = “Yes”** (conflict of interest disclosed).○**N1–N5 scores > 3** (ensuring methodological rigor).
**Include preselected studies** in the final synthesis.

#### Screening and Selection Process

**Figure 1:**
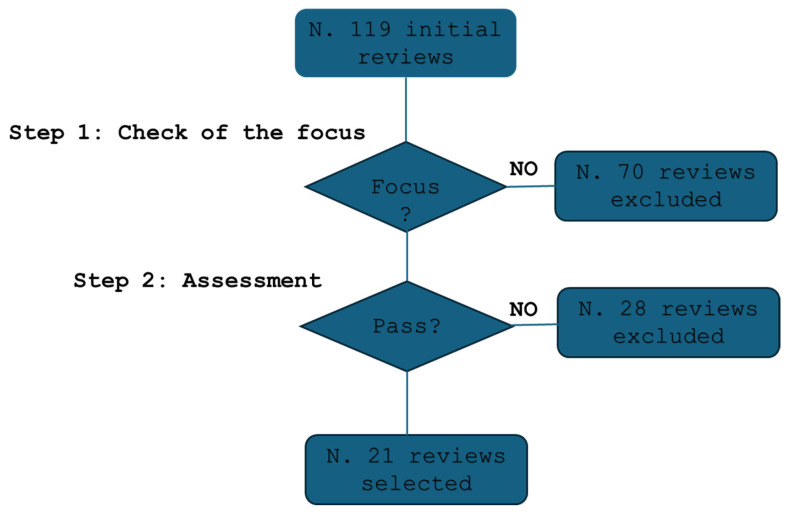
The flow of the selection process.

Figure 1 illustrates the selection process. An initial screening excluded 70 studies that lacked relevance or focused outside of cytopathology. A secondary qualification process further refined the selection, ensuring methodological rigor.

A total of 21 studies met all inclusion criteria and were selected for review [12,30,31,32,33,34,35,36,37,38,39,40,41,42,43,44,45,46,47,48,49].

### 2.3. Bibliometric Analysis Methodology

A bibliometric analysis was conducted using PubMed to complement the narrative review by assessing trends in digital cytopathology research. The analysis focused on the following:

- The historical trajectory of research in digital cytopathology.
- Research trends over the last 10 years.
- More recent trends in the last 5 years.

#### Trend Analysis

The analysis tracked the evolution of research output, detecting both original research articles and review articles over time. Special attention was given to the sharp increase in publications in recent years and the growing role of AI in digital cytopathology.

- The growth in AI-related research was analyzed, highlighting the increasing prevalence of machine learning and deep learning technologies.
- The bibliometric trends were presented in a narrative format with graphical representations, illustrating the progression of digital cytopathology research and the shift towards AI-driven innovations.

This methodology ensures a systematic approach to reviewing the digital cytopathology literature while capturing research trends over time.

## 3. Results

The results are structured into three distinct sections, each addressing a specific aspect of the analysis. Section 3.1 focuses on tracing the evolution of bibliometric trends within these fields, providing a comprehensive overview of how research activity and focus areas have developed over time. Section 3.2 delves into a detailed categorization of the studies, offering a systematic organization of the literature and presenting a unifying message that emerges from the analysis, shedding light on shared themes and conclusions. Lastly, Section 3.3 explores the opportunities and challenges identified through the analysis, discussing their implications and potential directions for future research and practice in the field.

### 3.1. Trends

A bibliometric analysis conducted on PubMed using the query (Cytopathology[Title/Abstract]) AND (Digital[Title/Abstract]) revealed key trends in digital cytopathology research.

**Figure 2:**
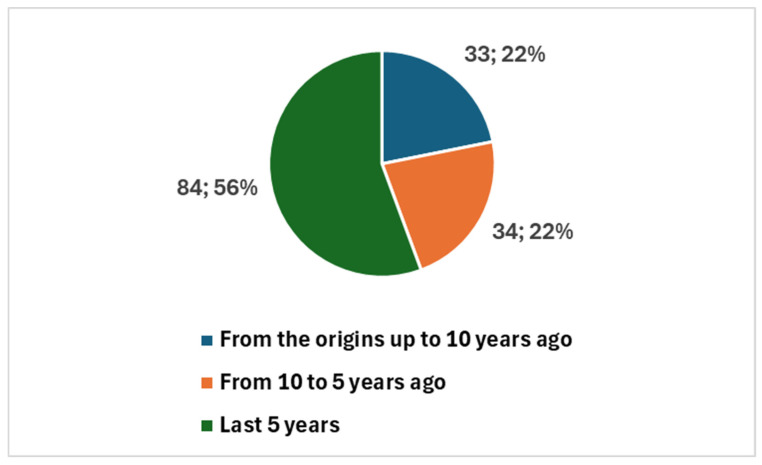
Trends in publications on digital cytopathology, referring to historical data, the last 10 years, and the last 5 years.

The earliest studies date back to 1984, with a total of 151 publications since then (Figure 2). Notably, 118 studies (78.1% of the total) were published in the last decade, with a sharp increase in the past five years, accounting for 84 publications (55.6%). This surge, particularly following the COVID-19 pandemic, reflects the accelerated adoption of digital tools in cytopathology to support remote diagnostics and maintain workflow continuity (Figure 2).

**Figure 3:**
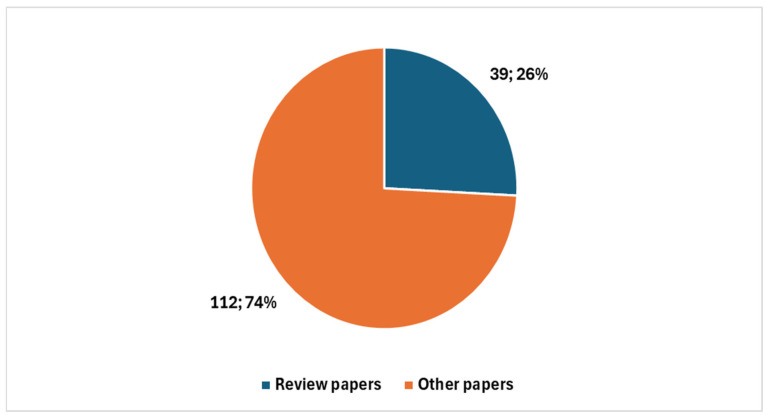
Proportion of studies on digital cytopathology, divided between reviews and other types.

**Figure 4:**
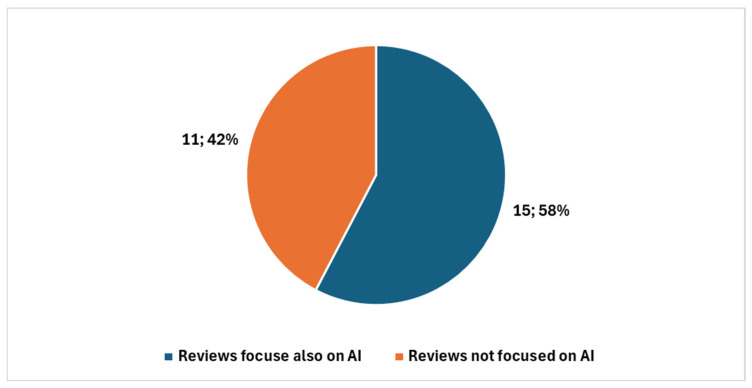
Reviews in digital cytopathology focused on AI and not focused on AI in the last five years.

Among these, 39 were review articles (Figure 3), with 26 published in the last five years. A significant portion of recent reviews—15 out of 26—focused specifically on artificial intelligence (AI) (Figure 4).

Applying an additional PubMed filter: (Cytopathology[Title/Abstract]) AND (Digital[Title/Abstract]) AND ((artificial intelligence[Title/Abstract]) OR (machine learning[Title/Abstract]) OR (deep learning[Title/Abstract])), we identified 32 AI-related studies since 1998, with 28 of them published in the last five years. This means that nearly half (46.9%) of all AI-related research in digital cytopathology has emerged recently, underscoring AI’s growing significance in the field.

The data highlight a clear trend: digital cytopathology research has expanded rapidly, with AI playing an increasingly central role. AI-driven technologies, particularly machine learning and deep learning, are being actively explored to enhance diagnostic accuracy, streamline workflows, and manage large datasets. The rise in AI-focused reviews suggests that researchers are not only investigating AI’s capabilities but also evaluating its integration into clinical practice. This shift aligns with the broader digital transformation of cytopathology, where AI is becoming a key enabler of innovation and efficiency.

### 3.2. Emerging Themes and Categorization

Digital tools and AI are revolutionizing cytopathology by improving diagnostic accuracy, workflow efficiency, and patient care. Advancements such as AI-driven chatbots and natural language processing (NLP) support data extraction, classification, and patient interactions, reducing errors and enhancing accessibility [30]. Machine learning (ML) applications are improving the diagnosis of thyroid and urothelial carcinoma and optimizing cytological workflows [33,43]. AI-powered image analysis further enhances diagnostic precision by identifying microscopic features with greater accuracy [31].

The integration of whole-slide imaging (WSI) in digital cytology offers significant improvements in diagnostics and training, with guidelines from the College of American Pathologists (CAP) emphasizing its validation for clinical use [12,46]. AI is also transforming microbiological diagnoses, particularly in resource-limited settings, by facilitating faster and more accurate microorganism identification [44]. Additionally, AI and next-generation sequencing contribute to personalized medicine by refining diagnostic capabilities and treatment strategies [33].

The COVID-19 pandemic accelerated the adoption of digital platforms, reinforcing the importance of AI in remote diagnostics, education, and cancer screening [42,45]. Digital cytology combined with immunocytochemistry is proving valuable for diagnosing metastatic breast carcinoma, while AI tools support the assessment of immune checkpoint inhibitors in lung cancer [40,41]. Furthermore, the role of AI-powered tools like ChatGPT in pathology workflows demonstrates their potential in assisting clinicians with cancer diagnosis and case management [39].

Despite these advancements, challenges such as AI validation, large dataset requirements, and ethical considerations remain [49]. The evolution of AI-driven tools, including convolutional neural networks (CNNs) and Z-stack scanning, continues to enhance diagnostic accuracy, particularly in complex cytological cases [47,48]. Overall, AI and digital technologies are reshaping cytopathology, offering unprecedented improvements in diagnostics, workflow optimization, and clinical decision making. As these innovations progress, they hold immense potential for advancing the precision and accessibility of cytopathological practice worldwide.

Table 1 provides a synthesis of these key contributions and their impact on the field [12,30,31,32,33,34,35,36,37,38,39,40,41,42,43,44,45,46,47,48,49]. The table summarizes various studies and technologies related to digital cytopathology, detecting also the key themes. Each reference is accompanied by a brief description of the study or technological advancement, highlighting its focus on digitalization and the emerging theme. This structured format helps reveal how AI and digital tools are shaping cytopathology and points to areas for future research and improvement.

Table 2 categorizes key advancements in digital cytopathology, providing a structured overview of emerging technologies and their contributions to the category. The table is organized into thematic areas, each highlighting specific innovations and their impact based on key technologies and approaches:

- AI-Powered Diagnostic Tools, covering AI-driven image analysis [31], workflow automation [37], and AI-assisted decision making [39], along with their applications in microbiological disease diagnosis [44] and thyroid cytology [43,47], can also enhance diagnostic accuracy, streamline workflows, and improve patient engagement, as highlighted in [30].
- Digital Pathology and Computational Techniques, addressing the role of digital cytology in molecular diagnostics [34], metastatic breast carcinoma [40], and lung cancer immunotherapy [41], as well as AI-powered whole-slide imaging (WSI) [38].
- Workflow and Efficiency Enhancements, focusing on preoperative cytology [32], computer-assisted diagnosis (CAD) for urothelial carcinomas [33], and technological advancements like Z-stack scanning [48] and EUS FNAB for pediatric pancreatic neoplasms [36].
- Impact of COVID-19 on Cytopathology, highlighting the role of remote diagnostics [45] and the challenges posed by the pandemic [42] in digital pathology adoption.
- Educational and Professional Development, discussing digital cytology for training [12], the impact of social media on professional networking [35], and standardization efforts through CAP guidelines [46].
- Ethical and Regulatory Considerations, exploring concerns related to AI ethics in digital cytology [49], including data privacy, regulatory compliance, and bias reduction.

This classification offers a comparative perspective on technological trends and ongoing challenges in cytopathology. By organizing studies in this way, the table facilitates a clearer understanding of how AI and digital tools are shaping the field while also identifying key areas for future research and standardization.

**Table 2:** Categorization of key advancements in digital cytopathology.

Thematic Category	Key Technologies and Approaches	Main Contributions	Ref.
AI-Powered Diagnostic Tools	Chatbots and NLP	Enhances data extraction, classification, and patient interaction	[30]
.	AI-driven image analysis	Increases diagnostic precision in cytopathology	[31]
.	AI-enhanced workflow automation	Improves diagnostic processes, reduces turnaround time	[37]
.	AI-assisted pathology decision making	Provides real-time diagnostic support to pathologists	[39]
	Machine learning for thyroid cytology	Improves classification of indeterminate cases	[43]
	AI for microbiological disease diagnosis	Enhances pathogen identification and infection diagnostics	[44]
	AI and WSI integration in thyroid cytology	Reduces uncertainty in indeterminate cases	[47]
Digital Pathology and Computational Techniques	Digital pathology for molecular diagnostics	Enhances precision medicine approaches	[34]
.	Digital cytology in metastatic breast carcinoma	Enables more precise and personalized treatment options	[40]
.	Digital cytology for PD-L1 assays	Supports lung cancer immunotherapy decisions	[41]
	AI-powered WSI	Improves diagnostic efficiency and accuracy	[38]
Workflow and Efficiency Enhancements	AI-assisted pathology workflows	Reduces human error and increases efficiency	[39]
	Z-stack scanning and AI	Enables precise cytological assessments	[48]
.	Preoperative cytology for salivary lesions	Enhances diagnostic accuracy for complex lesions	[32]
	CAD for urothelial carcinomas	Improves cancer detection accuracy and efficiency	[33]
	EUS FNAB for pediatric pancreatic neoplasms	Provides minimally invasive, high-accuracy diagnostics	[36]
Impact of COVID-19 on Cytopathology	Remote diagnostics and digital pathology	Enabled pathology practices to continue during the pandemic	[45]
.	Challenges in cytopathology during COVID-19	Highlighted infrastructure gaps and workflow disruptions	[42]
Educational and Professional Development	Digital cytology for pathology training	Enhances virtual learning and global knowledge exchange	[12]
.	Social media for professional networking	Encourages knowledge dissemination and collaboration	[35]
	CAP guidelines for WSI adoption	Establishes standardization for digital pathology	[46]
Ethical and Regulatory Considerations	AI ethics in digital cytology	Ensures transparency and accountability in AI applications	[49]

### 3.3. Opportunities and Areas Needing Further Improvements

#### 3.3.1. Opportunities

The integration of digital health into cytopathology presents numerous opportunities to enhance diagnostic accuracy, streamline workflows, and improve patient outcomes. These advancements span various domains, including AI-powered diagnostic tools, molecular diagnostics, workflow efficiency, professional development, and the application of new technologies in pediatric and rare disease diagnostics.

Table 3 below categorizes the key opportunities in cytopathology, highlighting how digital tools contribute to improved diagnostic precision, enhanced efficiency, and expanded professional collaboration and potential impact. These advancements also facilitate the adoption of personalized medicine approaches and optimize cytopathology workflows. By addressing existing challenges and leveraging these opportunities, the field can move toward more reliable and accessible diagnostic solutions.

Key areas of opportunity include the following:

- AI and Machine Learning Integration in Diagnostic Accuracy—AI-driven tools enhance diagnostic accuracy through natural language processing, image analysis, and workflow automation. These technologies assist pathologists in decision making, reducing human error and improving efficiency.
- Digital Pathology and Technology in Molecular Diagnostics—Digital pathology complements molecular investigations, enabling more precise biomarker analysis and targeted treatment strategies, particularly in cancer diagnostics.
- Improved Diagnostic Workflow and Efficiency—Digital cytopathology facilitates workflow enhancements, increasing diagnostic speed and reducing turnaround times, particularly in the classification of complex cytology cases.
- Professional Development and Collaboration—The adoption of digital tools and social media enhances knowledge sharing, professional networking, and educational opportunities in cytopathology.
- Advancements in Pediatric and Rare Disease Diagnostics—AI and digital cytopathology provide innovative solutions for diagnosing rare and pediatric conditions, offering non-invasive and highly accurate methodologies.
- Impact of the COVID-19 Pandemic—The pandemic accelerated the adoption of digital pathology and remote diagnostic solutions, highlighting the potential for increased flexibility in pathology practice.

#### 3.3.2. Challenges

While digitalization offers significant advancements in cytopathology, several challenges must be addressed to ensure its successful integration into clinical practice. These challenges span multiple domains, including data management, regulatory concerns, technological limitations, clinical application hurdles, and barriers to widespread adoption.

Table 4 below categorizes the primary obstacles that hinder the seamless implementation of digital tools in cytopathology with the potential impact. These challenges range from data standardization issues and regulatory complexities to the need for robust validation frameworks and infrastructure improvements. Additionally, ethical concerns, integration difficulties, and resistance to change present further barriers that must be overcome.

Key areas of challenges include the following:

- Data, AI, and Integration Challenges—Issues related to data standardization, AI biases, and integration difficulties in clinical workflows pose significant barriers to AI adoption in cytopathology.
- Regulatory, Validation, and Ethical Concerns—The need for regulatory approvals, ethical considerations, and robust validation frameworks remains a critical challenge in ensuring the reliability and safety of AI-driven diagnostics.
- Technological and Infrastructure Limitations—High implementation costs, computational requirements, and infrastructure gaps in resource-limited settings hinder the widespread use of digital pathology and AI tools.
- Clinical and Diagnostic Application Challenges—Variability in diagnostic accuracy, lack of standardized protocols for emerging biomarkers, and challenges in pediatric and rare-disease diagnostics limit the effectiveness of AI applications in cytopathology.
- Adoption and Practice Integration Barriers—Resistance to transitioning from traditional cytology methods, along with the need for specialized training and compliance with professional guidelines, presents obstacles to widespread AI adoption.

By systematically addressing these challenges, the field of cytopathology can work toward developing more reliable, accessible, and standardized AI-driven solutions.

**Table 4:** Categorization of challenges.

Thematic Category	Key Challenges	Impact on Implementation	Ref
Data, AI, and Integration Challenges	Data standardization issues in AI systems, particularly in cytology compared to histology and radiology.	Hinders interoperability and consistency in AI-driven diagnostics.	[30]
Data, AI, and Integration Challenges	Biases in AI models, lack of clinical validation, and ethical/privacy concerns.	Raises trust issues and regulatory hurdles for AI adoption.	[30]
Data, AI, and Integration Challenges	Early image analysis limitations in computational power and biological sample complexity.	Slows down AI integration into clinical workflows.	[31]
Data, AI, and Integration Challenges	Lack of peer-reviewed, real-world data and integration challenges in clinical settings.	Creates barriers to AI adoption in cytology diagnostics.	[37]
Data, AI, and Integration Challenges	Potential biases in AI models and legal concerns regarding AI-based diagnoses.	Limits clinical adoption due to reliability concerns.	[39]
Data, AI, and Integration Challenges	Need for large, diverse datasets for AI training and technical limitations in scanning cytology specimens.	Reduces AI effectiveness and applicability in clinical settings.	[43]
Data, AI, and Integration Challenges	Limited AI application in cytology due to integration challenges and computational resource requirements.	Slows down AI-driven advancements in cytopathology.	[48]
Data, AI, and Integration Challenges	Need for robust AI validation and integration with whole-slide imaging (WSI).	Affects diagnostic precision and standardization.	[47]
Regulatory, Validation, and Ethical Concerns	Regulatory approval and validation challenges hinder AI adoption in clinical practice.	Delays implementation of digital pathology solutions.	[33]
Regulatory, Validation, and Ethical Concerns	Ethical and privacy concerns, including risks of misinformation and inadequate data protection.	Requires strict regulatory frameworks for safe AI deployment.	[35]
Regulatory, Validation, and Ethical Concerns	Standardization issues for emerging biomarkers and digital cytopathology validation.	Slows down the development of innovative diagnostic tools.	[40]
Regulatory, Validation, and Ethical Concerns	Limited validation of immunotherapy markers and challenges in standardizing imaging techniques.	Creates inconsistencies in immunotherapy testing.	[41]
Regulatory, Validation, and Ethical Concerns	Data security and cybersecurity concerns related to large AI training datasets.	Increases risks of data breaches and regulatory non-compliance.	[49]
Technological and Infrastructure Limitations	AI algorithm limitations, lack of annotated datasets, and scalability concerns.	Restricts AI applications in low-resource settings.	[44]
Technological and Infrastructure Limitations	Lack of digital cytopathology infrastructure and resistance to change from glass slide methods.	Slows adoption and transition to digital workflows.	[45]
Technological and Infrastructure Limitations	High initial costs and specialized infrastructure needs for digital cytology.	Limits widespread implementation in pathology labs.	[12]
Technological and Infrastructure Limitations	Large file sizes and increased acquisition times for whole-slide images.	Requires substantial computational resources and storage capacity.	[49]
Clinical and Diagnostic Application Challenges	Heterogeneous lesion interpretation and preoperative challenges in salivary gland tumors.	Limits diagnostic accuracy for complex conditions.	[32]
Clinical and Diagnostic Application Challenges	Validation issues for new biomarkers and standardization of protocols.	Creates inconsistencies across diagnostic systems.	[40]
Clinical and Diagnostic Application Challenges	Limited availability of specialized diagnostic tools for pediatric cytopathology.	Delays advancements in pediatric AI applications.	[36]
Clinical and Diagnostic Application Challenges	COVID-19-related diagnostic delays and decline in cancer screenings.	Highlights accessibility and efficiency issues in cytopathology.	[42]
Adoption and Practice Integration Barriers	Limited digital cytopathology implementation compared to histopathology.	Slows down integration into routine practice.	[45]
Adoption and Practice Integration Barriers	Challenges in AI tool validation and compliance with CAP guidelines.	Hinders standardization and acceptance of digital pathology.	[46]
Adoption and Practice Integration Barriers	Resistance to transition from glass slides to digital platforms.	Increases barriers to adopting AI-driven diagnostic methods.	[12]

## 4. Discussion

The discussion is organized into five comprehensive sections. The Section 4.1 reports a synoptic of the organization of the discussion, detailing the rationale based on the output of the results.

Section 4.2 presents the key evidence derived from the overview of reviews, with a particular emphasis on detailing the added value they provide to the field. Section 4.3 focuses on the emerging recommendations that arise from the analysis, offering insights into best practices and potential strategies for further development. Section 4.4 shifts the focus to recent primary studies, analyzing their findings and perspectives in light of the emerging recommendations to assess their alignment and relevance. Section 4.5 focuses on and discusses, in light of the emerging findings, the significant potential future developments related to the integration of AI with digital processes in this field. Lastly, Section 4.6 provides a critical evaluation of the review, outlining its limitations and discussing areas for improvement in future research efforts.

### 4.1. Synoptic Overview of the Study Rationale

**Figure 5:**
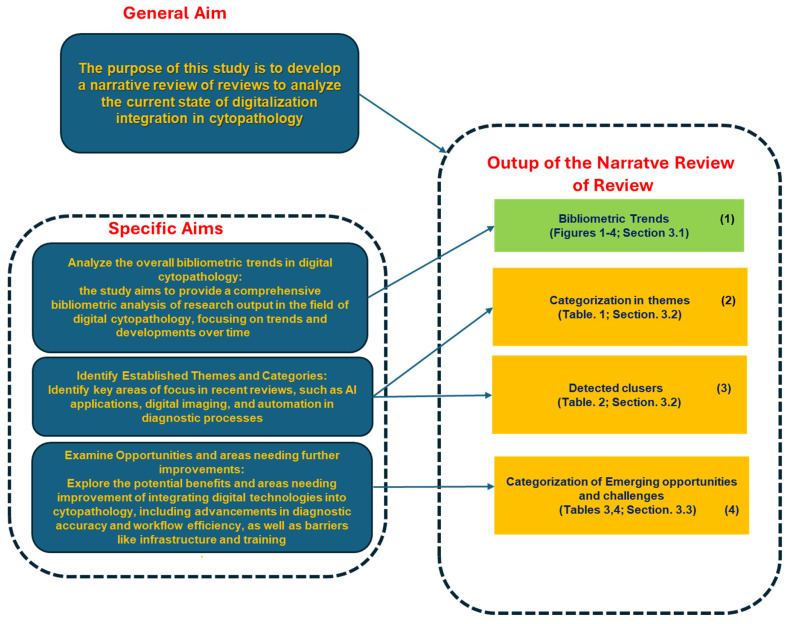
First synoptic diagram.

**Figure 6:**
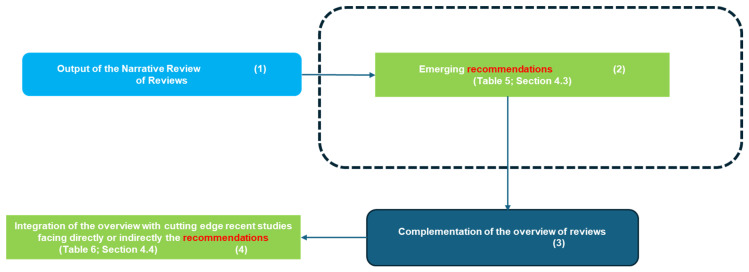
Second synoptic diagram.

Figure 5 and Figure 6 present two synoptic diagrams that provide a structured visual representation of the design and rationale behind the narrative review of reviews (NRR). These diagrams illustrate the logical sequence of the study’s phases and their interconnections.

#### 4.1.1. First Diagram (Figure 5): Linking Objectives to Analysis

The first synoptic diagram (Figure 5) outlines how the study was structured based on its overall objective and three specific objectives. The logical flow follows a top-down approach:

- Block 1 (Top): This block represents the bibliometric trends reported in Figure 2, Figure 3 and Figure 4 (Section 3.1). These trends were analyzed to provide an overview of the scientific production on digital cytopathology, with a focus on AI contributions and historical evolution over the past 10 and 5 years.
- Block 2 (Upper Middle): This block corresponds to the categorization of studies by thematic areas, as presented in Table 1 (Section 3.2). The classification helped structure the reviewed studies based on key themes, digitalization focus, and study descriptions.
- Block 3 (Lower Middle): Building on the thematic categorization, this block highlights the comparative side-by-side analysis of the studies. The classification into clusters, as reported in Table 2 (Section 3.2), is structured according to Thematic Category, Key Technologies and Approaches, and Main Contributions.
- Block 4 (Bottom): This block synthesizes the opportunities and challenges identified in the reviewed studies, as detailed in Table 3 and Table 4 (Section 3.3). These findings, organized in clusters under the fields Thematic Category/Challenge, Key Opportunities/Challenges, and Potential Impact, highlight both the benefits of AI applications—such as workflow optimization and improved diagnostic accuracy—and the challenges related to standardization, ethics, technological barriers, and accessibility.

This synoptic diagram provides a clear, step-by-step visualization of the study’s methodological process, from bibliometric analysis to thematic categorization, comparative assessment, and the identification of emerging opportunities and challenges.

#### 4.1.2. Second Diagram (Figure 6): Connecting Findings to Recommendations

The second synoptic diagram (Figure 6) is logically connected to the first and illustrates how the study transitions from literature review findings to discussion and recommendations. The sequential structure follows a well-defined approach:

- Block 1 (Top Left): This block represents the findings derived from the reviewed studies, forming the basis for the discussion (Section 4.3).
- Block 2 (Top Right): This block presents the recommendations extracted from the review, as summarized in Table 5 (Section 4.3). These recommendations are directly connected to the insights gathered from the reviewed studies.
- Block 3 (Bottom Right): Highlights the need to complement the overview with cutting-edge primary studies to assess how recent advancements align with the recommendations identified in the review.
- Block 4 (Bottom Left): Refers to Table 6 (Section 4.4), which groups the most recent cutting-edge studies into clusters based on Related Categories, Study Number, Key Findings, Takeaways, and Relevant Recommendations.

Together, these synoptic diagrams provide a clear and structured overview of the study’s rationale. Figure 5 outlines the transition from bibliometric analysis to thematic categorization and comparative assessment, leading to the identification of key opportunities and challenges. Figure 6 extends this framework by illustrating how these findings translate into practical recommendations and align with recent cutting-edge research.

This structured representation ensures clarity and coherence, making explicit the connections among different research phases and highlighting the relevance of AI applications, digitalization challenges, and future directions in digital cytopathology.

### 4.2. Highlights from the Overview

This narrative review provides a comprehensive analysis of reviews on the digitalization of cytopathology, highlighting key trends, challenges, and opportunities in this evolving field. It makes several significant contributions to the literature.

First, it identifies emerging themes shaping digital cytopathology. Approximately 50% of the 18 reviews analyzed emphasize AI’s transformative role in cytopathology [30,31,33,34,37,38,39,43,44]. Notably, AI-driven technologies such as large language models (LLMs) and chatbots [30,39] show promise in assisting pathologists with real-time support and complex diagnostics, though they raise concerns about ethics, data privacy, and algorithmic bias.

Second, the reviews highlight future directions for digital cytopathology, emphasizing AI and digital tools as catalysts for faster, more precise, and accessible diagnostics. Digital platforms can also enhance global collaboration, particularly in resource-limited settings.

Third, two reviews [37,38] provide targeted recommendations for AI adoption, stressing the importance of whole-slide imaging, standardized AI protocols, and workforce training. Robust validation processes are essential to ensure reliability, address biases, and build trust in AI-driven diagnoses. Cross-disciplinary collaboration between AI experts, pathologists, and regulatory bodies is also critical for maintaining high-quality standards.

Finally, digital cytopathology has the potential to bridge healthcare gaps, particularly in remote areas. AI-driven diagnostics can reduce reliance on physical consultations, improving healthcare accessibility and outcomes.

**Figure 7:**
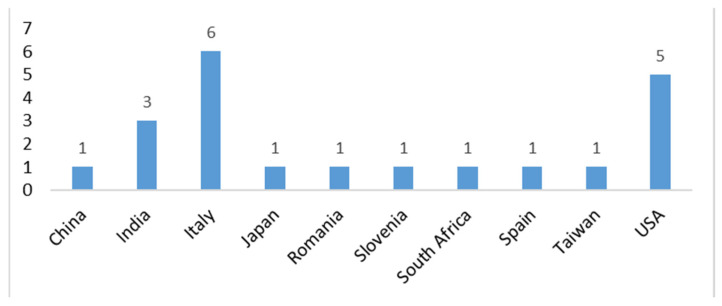
Origin of the study based on the corresponding author.

Overall, this study underscores AI’s pivotal role in digital cytopathology, balancing its opportunities with ethical, regulatory, and implementation challenges. The insights from these reviews serve as a roadmap for successfully integrating AI into clinical practice. Figure 7 illustrates the origin of the study based on the corresponding author.

### 4.3. Emerging Recommendations

Studies on digital cytopathology provide a comprehensive view of the field, offering key recommendations for advancing these technologies. While Kim et al. [37,38] provide in-depth guidelines, other reviews also highlight essential insights for successful AI adoption in cytopathology.

A central theme is the need for standardization, ensuring AI tools and digital cytology techniques like whole-slide imaging (WSI) are consistently applied across laboratories [30,33,37]. Standardized datasets and validation protocols enhance AI reliability across clinical settings.

Training and education for cytopathologists and cytotechnologists are also crucial [31,34]. AI integration requires not just technical skills but also knowledge of ethical, legal, and regulatory considerations. Training should emphasize AI as a diagnostic support tool rather than a replacement for human expertise.

The studies stress rigorous validation of AI tools before clinical deployment [33,44]. AI applications must be tested in real-world settings with diverse patient data to ensure accuracy. Continuous monitoring and iterative refinement further improve AI performance [37].

Kim et al. [37] also highlight the need for legal frameworks addressing data privacy, patient consent, and AI accountability. Collaboration among clinicians, regulatory bodies, and AI experts is necessary to mitigate biases and enhance AI trustworthiness.

Beyond regulatory concerns, reviews explore AI’s role in enhancing diagnostics. AI-driven tools such as convolutional neural networks (CNNs) show promise in improving accuracy for complex cases such as thyroid lesions [47]. AI also facilitates molecular analysis in cytology, enabling faster and more precise cancer detection [48].

Infrastructure challenges, such as large file sizes and computing demands, must be addressed to ensure AI accessibility in clinical practice [49]. Standardizing digital slide preparation and stain integration is essential for maintaining data quality.

On a global scale, AI and digital cytology can improve diagnostic access in underserved regions, enabling telepathology and international collaboration [30,33,37,38].

In conclusion, Kim et al. [37,38] provide critical and specific broad recommendations for AI adoption, and the broader literature underscores the importance of standardization, training, validation, and collaboration. Implementing these strategies will enhance diagnostic accuracy, efficiency, and global accessibility in cytopathology.

Table 5 resumes the key emerging recommendations.

**Table 5:** Emerging recommendations.

	Recommendation	Description	References
1	Standardization of Protocols	Implement standardized protocols for digital cytology and AI integration, ensuring consistency in scanning, data handling, and AI application across various clinical settings.	[30,33,37,47]
2	Comprehensive Training and Education	Provide training for cytopathologists and cytotechnologists on AI tools and digital workflows, covering both technical and ethical aspects to ensure effective use in diagnostics.	[31,34,37,48]
3	Collaborative Development of AI Tools	Facilitate collaboration among pathologists, AI experts, and regulatory bodies to develop AI tools that meet high clinical, ethical, and safety standards.	[37,38,44,49]
4	Continuous Validation of AI Systems	Continuously validate AI algorithms in real-world clinical settings, incorporating peer-reviewed studies and regular updates to improve performance.	[33,37,44,47]
5	Legal and Ethical Considerations	Establish guidelines addressing transparency, patient consent, data security, and bias prevention in AI, ensuring ethical and lawful AI implementation.	[37,38,48]
6	AI Integration into Workflows	Integrate AI into clinical workflows to assist pathologists in routine tasks, improving diagnostic accuracy and efficiency without replacing human expertise.	[37,38,44,49]
7	Global Collaboration and Knowledge Sharing	Foster international collaboration to share knowledge and best practices, expanding access to digital cytology and AI, particularly in underserved areas.	[30,33,37,47]
8	Increased Accessibility in Remote Areas	Leverage digital cytology and AI to provide diagnostic services in remote regions, reducing the need for long-distance travel and enhancing healthcare access.	[30,33,37]

### 4.4. Key Contributions of Cutting-Edge Research in Advancing Digital Cytopathology

In addition to the overview of reviews, we have analyzed further primary studies that follow the trends and recommendations identified in the reviews. These studies provide a cutting-edge exploration of the latest advancements in digital cytopathology, highlighting innovative approaches that align with the broader trend of integrating technology into diagnostic practices [50,51,52,53,54,55,56,57,58,59,60,61,62,63,64,65,66,67,68,69]; see Table 6.

These primary research studies provide valuable insights into the rapidly evolving landscape of digital cytology, reflecting ongoing efforts to improve diagnostic accuracy, enhance workflow efficiency, and address challenges in both routine practice and education. The following are the emerging themes with the relevant recommendation directly or indirectly addressed, with a brief justification in parentheses:

1. Whole-Slide Imaging (WSI) in Cytopathology and AI Validation

Whole-slide imaging has emerged as a reliable alternative to traditional light microscopy for cytological evaluations. However, several studies highlight the challenges of resolving borderline cases (e.g., ASC-US, ASC-H) and the potential for misdiagnosis in nuanced cases, necessitating further technological refinements to make WSI a routine diagnostic tool [50,52]. Relevant recommendations: The need for enhanced diagnostic accuracy and workflow integration aligns with recommendations 1 (WSI standardization) and 4 (technological improvements in digital cytopathology).

2. AI-Assisted Digital Cytopathology and AI Integration

The integration of artificial intelligence (AI) in cytology, particularly through systems such as the Hologic Genius Digital Diagnostics System (GDDS) and CytoGAN, is revolutionizing diagnostic practices by enhancing sensitivity and accuracy in detecting lesions, particularly for challenging conditions like high-grade squamous intraepithelial lesions (HSIL) and endometrial cancer. AI models like AIxURO for bladder cancer also demonstrate significant improvements in diagnostic efficiency, reducing screening times while improving sensitivity [51,53,54,55]. Relevant recommendations: The importance of AI-assisted diagnostics and its integration into routine practice is addressed in recommendations 6 (AI standardization and validation) and 4 (technological advancements for improved performance).

3. Telecytology for Remote Evaluation and AI in Remote Access

Telecytology is proving to be a valuable tool, especially in the context of rapid on-site evaluation (ROSE) and remote consultations, addressing challenges posed by cytotechnologist shortages and the need for timely diagnoses. Studies validating telecytology systems highlight their effectiveness in maintaining high diagnostic quality, even in remote or resource-limited environments [56,59,66]. Relevant recommendations: These findings reinforce recommendations 7 (telecytology standardization) and 8 (expanding accessibility of digital cytology in low-resource settings).

4. AI Adoption and Workflow Integration Challenges

Despite the promising potential of digital cytology and AI, challenges remain in their widespread adoption. Studies reveal that while WSI is increasingly used in surgical pathology, its use in cytology lags behind, partly due to concerns over image quality and the cost of implementation. The integration of AI into routine practice is also slow, reflecting the need for further advancements in both technology and practical guidelines [57]. Relevant recommendations: This aligns with recommendations 1 (encouraging WSI adoption) and 6 (AI implementation strategies).

5. Innovative Scanning Approaches and AI Optimization

Novel scanning techniques, such as AI-assisted heuristic scanning, present alternatives to traditional multi-Z-plane methods, offering faster, more cost-efficient approaches for digitizing cytology slides without compromising diagnostic accuracy. These approaches are pivotal in optimizing the scanning process for conditions like urothelial carcinoma [58]. Relevant recommendations: This supports recommendations 1 (digital cytology optimization) and 6 (improvements in AI-driven scanning technologies).

6. COVID-19 Impact on Digital and Telecytology Practices

The COVID-19 pandemic has catalyzed the adoption of digital technologies and telecytology, particularly for remote evaluations. While the overall usage of digital cytology remained stable, the pandemic significantly increased the use of telecytology for ROSE, highlighting its growing role in improving diagnostic workflows during public health crises [59]. Relevant recommendations: This is aligned with recommendations 7 (strengthening telecytology applications) and 8 (ensuring accessibility in crisis situations).

7. Educational Advancements through Digital Platforms and AI in Training

Beyond diagnostics, digital platforms are playing a crucial role in enhancing education and mentorship in cytopathology. Virtual journal clubs and e-learning modules, such as those for thyroid cytopathology, are expanding global access to training, offering valuable resources for both trainees and practicing pathologists [60,64,69]. Relevant recommendations: The role of digital platforms in education is directly linked to recommendations 2 (expanding virtual education) and 7 (integrating AI-driven learning tools).

8. Affordable and Portable Solutions for Cytology in Resource-Limited Settings

The introduction of low-cost, portable devices like the Landing-Smart scanner addresses a critical gap in underdeveloped regions, enabling high-quality cytology screening without the financial burden of traditional WSI systems [68]. Similar innovations during the COVID-19 pandemic demonstrated the potential of cost-effective solutions in bridging diagnostic disparities [62]. Relevant recommendations: These studies support recommendations 8 (expanding digital cytology in underserved areas) and 6 (ensuring AI accessibility in low-resource settings).

9. Other Significant Advancements in Digital Cytology

- Digital Cytology Validation: The use of deep learning for enhancing lung cancer diagnosis has demonstrated promising results, improving diagnostic precision and reducing interobserver variability [63].
- Scanner Performance Comparisons: Studies evaluating the performance of different digital cytology scanners highlight the importance of multi-layer Z-stacking to enhance atypical cell detection [61].
- Rapid Online Evaluation: Research on real-time assessments for endoscopic cytology specimens emphasizes the efficiency gains from telecytology-based rapid evaluations [65].
- Glioma Diagnosis in Digital Cytology: Preliminary investigations into the validation of digital cytology for glioma diagnoses indicate high concordance rates, reinforcing its potential as a diagnostic tool [67]. Relevant recommendations: These studies address recommendations 1 (improving scanner technology), 4 (advancing digital pathology), 6 (validating AI applications), and 7 (enhancing real-time telecytology).

Overall, these studies reflect a dynamic shift towards more efficient, accurate, and accessible cytological diagnoses, underscoring the transformative potential of digital pathology, AI, and telemedicine in shaping the future of cytopathology. As these technologies continue to evolve, they promise to enhance diagnostic workflows, expand educational opportunities, and address the growing demands of a globalized healthcare system.

Table 6 provides a summary of the key findings in cutting-edge recent studies, their categorization, and the indirectly addressed recommendations.

**Table 6:** Sketch of the key findings in cutting-edge recent studies, along with the direct or indirect connection with the faced recommendations.

Related Categories	Study Number	Key Findings	Takeaway	Relevant Recommendation(s)
Whole-Slide Imaging in Cytopathology, AI Validation	[50]	Compared WSI and conventional light microscopy (CLM) in thin-layer cervical samples. High agreement in NILM categories but lower agreement in borderline cytological categories (ASC-US, ASC-H).	WSI is reliable for many categories but requires improvement for borderline lesions.	1, 4
Whole-Slide Imaging in Cytopathology, AI Validation	[52]	Investigated WSI for intraoperative touch imprint cytology of sentinel lymph nodes (SLNs) in breast cancer patients. High concordance rates but slight accuracy reductions compared to light microscopy.	WSI is feasible for intraoperative evaluation but needs technological refinement to reduce misdiagnosis risks.	1, 4
AI-Assisted Digital Cytopathology, AI Integration	[51]	Evaluated the Hologic Genius Digital Diagnostics System (GDDS) for AI-assisted diagnosis of HSIL. Excellent sensitivity (84.7–92.9%) and strong interobserver agreement (Kendall W = 0.722).	AI-assisted systems like GDDS significantly enhance HSIL diagnosis with strong agreement and sensitivity.	6, 4
AI-Assisted Digital Cytopathology, AI Development	[53]	Introduced CytoGAN, a deep-learning model for realistic stain transfer in cytopathology images. Improved endometrial cancer classification by 20%.	AI-based stain transfer models improve consistency and accuracy in image analysis, critical for multimodal datasets.	6, 4
AI-Assisted Digital Cytopathology, AI Integration	[54]	Tested AIxURO, an AI-enhanced urine cytology tool for bladder cancer. AIxURO improved sensitivity (from 30.6% to 63.9%) and reduced screening times by up to 83.2%.	AI platforms like AIxURO optimize diagnostic accuracy and efficiency in bladder cancer cytology.	6, 4
AI-Assisted Digital Cytopathology, AI Enhancement	[55]	Proposed STAR-RL, a reinforcement learning framework for pathology image super-resolution. Enhanced recovery of pathology images, improving diagnostic accuracy.	Super-resolution techniques address resolution limitations, improving diagnostic precision.	6, 4
Telecytology, Remote AI Access	[56]	Validated a cost-effective telecytology system using digital cameras and Microsoft Teams for ROSE in fine-needle aspiration samples. Achieved >90% adequacy assessment concordance.	Telecytology provides a practical solution for remote adequacy assessments, improving workflow efficiency.	7, 8
AI Adoption, Workflow Integration	[57]	Global survey on WSI and AI implementation in surgical pathology and cytology. Adoption in cytology lags behind surgical pathology due to challenges in cost and image quality.	Digital cytology adoption lags, with challenges in image quality and AI integration.	1, 6
Novel Scanning Approaches, AI Optimization	[58]	Introduced AI-based heuristic scanning as an alternative to multi-Z-plane scanning for urine cytology slides. Achieved similar cell capture rates while reducing scanning times and file sizes.	Heuristic scanning offers a faster, more cost-efficient approach for urine cytology digitization.	1, 6
Telecytology, Pandemic Impact	[59]	Survey comparing digital cytology practices pre- and post-COVID-19. Telecytology for ROSE increased significantly.	COVID-19 accelerated telecytology adoption, highlighting the need for validation and competency guidelines.	7, 8
Virtual Education, AI in Training	[60]	Described a two-year virtual journal club in gynecologic pathology. Enhanced global trainee engagement and mentorship.	Virtual journal clubs expand educational outreach and improve skills for trainees and pathologists.	2, 7
Digital Cytology, Scanner Performance	[61]	Compared Leica Aperio AT2 (Leica Biosystems, Nussloch, Germany) and Hamamatsu NanoZoomer S360 scanners (Hamamatsu photonics, Hamamatsu, Japan) for urine cytology slides. Optimal focus settings and Z-stacking improve a typical cell detection.	Optimal scanner focus and multi-layer Z-stacking enhance atypical cell detection but increase scanning time and file size.	1, 4
Cytopathology in Resource-Limited Settings	[62]	Analyzed the impact of COVID-19 on cytopathology in resource-limited regions. Digital resources and workflow modifications ensured continuity.	Digital resources were crucial for maintaining cytopathology services in resource-limited areas during the pandemic.	7, 8
Deep Learning for Cancer Diagnosis	[63]	Developed a deep-learning model to improve lung cancer diagnosis in respiratory cytology. Achieved 95.9% sensitivity and outperformed pathologists.	Deep learning models enhance diagnostic accuracy and reduce inter-observer variability in lung cancer cytology.	6, 4
Virtual Microscopy, Cytology Education	[64]	Evaluated a virtual microscopy platform for nongynecological cytology education. Virtual microscopy showed lower accuracy than light microscopy but received mixed feedback from students.	Virtual microscopy shows promise in education but requires improvements in image quality and platform performance.	2, 7
Telecytology, Rapid Online Evaluation	[65]	Assessed rapid online evaluation of endoscopically obtained cytological specimens. Achieved high sensitivity and specificity, especially in FNAs.	Rapid online evaluation improves sensitivity and allows for real-time sample adequacy.	7, 8
Telecytology, Remote ROSE Evaluation	[66]	Compared telecytology ROSE to traditional ROSE for lymph node and thyroid FNAs. Telecytology improved adequacy for lymph node FNAs.	Telecytology ROSE improves sample adequacy for complex cases, optimizing workflow and diagnostic quality.	7, 8
Digital Cytology Validation	[67]	Validated digital scanning of cytology specimens using the Leica Aperio GT 450 system. Achieved 98.7% concordance between digital and conventional diagnoses.	Digital cytopathology offers high diagnostic concordance, but optimization is needed for poorly cellular and thick samples.	1, 4
Portable Pathology Scanners, AI Accessibility	[68]	Introduced Landing-Smart, a low-cost, portable scanner for cytopathology. Demonstrated comparable accuracy to general digital scanners for cervical cytology specimens.	Landing-Smart provides a cost-effective, portable solution for cytology screening in resource-limited areas.	8, 6
AI in Education, E-Learning Modules	[69]	Developed a 35 min e-learning module to teach cytologic–histologic correlation in thyroid cytopathology. High satisfaction from participants.	The digital module enhances cytology–histology learning and is highly valued by students and residents.	2, 7

### 4.5. Digital Cytopathology Meets AI: Next Steps

#### 4.5.1. The Potential of AI

This NRR focused on the integration of digital health in cytopathology clearly highlights, both from the themes emerging from the review studies and the bridge with cutting-edge primary studies, the growing contribution of AI in this area. Although this narrative review is not specifically aimed at this field, which is addressed in other contributions, it is important to emphasize the emerging trends in order to outline the next steps. Tummala et al. [70], in the digital pathology domain, addressed the limitations of traditional diagnostic methods for lung and colon cancer, which, while still the gold standard, require significant time for analysis and high-end equipment and are prone to inter-observer variability. To overcome these challenges, they developed an automated method using AI-based EfficientNetV2 models, an advanced deep learning architecture grounded in compound scaling and progressive learning, applied to histopathological images from the LC25000 dataset.

Through rigorous cross-validation and five-fold testing, their model achieved performance that outperforms existing methods by a substantial margin. The results included an accuracy of 99.97%, an AUC of 99.99%, an F1-score of 99.97%, a balanced accuracy of 99.97%, and a Matthew’s correlation coefficient of 99.96%—all metrics that outperform traditional diagnostic techniques and highlight the superiority of AI-driven methods in this context.

The integration of Grad-CAM further enhanced the method by generating visual saliency maps, which pinpointed areas in histopathological images where the model focused its attention during predictions. These maps provide valuable insight and increase the model’s explainability, which is essential for clinical use. With these advancements, the proposed pipeline not only outperforms current methods but also presents a fully automated system that can be implemented in clinical settings. The ability of AI to outperform existing diagnostic systems offers pathologists a more efficient, reliable, and reproducible way to detect and diagnose lung and colon cancer, ultimately leading to improved treatment strategies and patient outcomes.

Staying within the digital pathology domain and focusing on cytopathology, several studies have shown AI’s potential to improve diagnostic performance [71]. For instance, AI can enhance the sensitivity and specificity of lung cancer diagnosis, which traditionally shows low performance in cytopathology. Sensitivity ranges from 0.49 to 0.71 for exfoliative sputum cytology and from 0.43 to 0.59 for abrasive cytology from bronchoscopy [63,72,73]. A systematic review of 26 studies on AI in non-gynecological cytopathology highlighted malignancies like thyroid, bladder, lung, breast, pancreas, ovary, and prostate. Thakur et al. showed that deep learning (DL) models achieved nearly 100% accuracy in analyzing 908 whole-slide images (WSI) for papillary thyroid carcinoma (PTCA), benign thyroid nodules (BTN), follicular adenoma (FA), and follicular carcinoma (FC). In breast cytopathology, an artificial neural network (ANN) model achieved 100% sensitivity and specificity when analyzing 112 image patches for fibroadenoma vs. invasive ductal carcinoma [74]. Wu et al. trained an AlexNet model using 85 WSIs from FNAC, achieving 78.20% accuracy in classifying histological subtypes, such as serous carcinoma, mucinous carcinoma, endometrioid carcinoma, and clear cell carcinoma [75]. For pancreas and prostate FNAC, accuracy and sensitivity were around 80%. These AI methods address cytology’s limitation that, while cost-effective, it will often have lower diagnostic accuracy. Thakur et al. noted that although cytology has advantages over histology, challenges in AI modeling arise due to annotation difficulties. In another study, 81,000 cervical cytopathological smear samples were collected from medical centers for annotating over 1.7 million images. An Xception model and decision tree were used to extract features, and AIATBS, an AI-assisted diagnostic solution, achieved rapid analysis (<180 s/smear) with 82.14% specificity and >83.00% sensitivity, adaptable to various staining, scanning, and sample preparation methods [76]. The review in [71], in addition to the examples mentioned above, highlights numerous studies across various cancer and pathology fields where AI models have demonstrated remarkable performance improvements. In brief, for lung cancer, the AlexNet model and DenseNet architecture have significantly enhanced diagnostic accuracy. In cervical cancer, AI models like Xception and AIATBS have achieved high specificity and sensitivity, enabling faster analysis with high diagnostic precision. The use of CellaVision and Morphogo in bone marrow aspirates and BMA smears, respectively, has also shown notable improvements in diagnostic performance. These AI-driven advancements have led to increased accuracy, with several models achieving near-perfect sensitivity and specificity, thereby demonstrating the potential of AI to revolutionize cancer diagnostics.

#### 4.5.2. The Next Steps

The ability of AI to outperform existing diagnostic systems offers pathologists a more efficient, reliable, and reproducible way to detect and diagnose various cancers and pathologies, ultimately leading to improved treatment strategies and patient outcomes.

Moving forward, integrating AI within the digital cytopathology field promises even more transformative advances. Emerging studies have already demonstrated AI’s significant role in improving diagnostic accuracy across various cancers and pathologies, including lung, cervical, breast, thyroid, bladder, and pancreatic cancers, as well as hematological conditions like acute myeloid leukemia and bone marrow disorders. These sectors merit substantial attention and further development. Future efforts should prioritize refining AI models to achieve higher precision and broader applicability across these diverse malignancies and pathologies.

Key to this progression will be enhancing the integration of AI-assisted diagnostic solutions, such as AIATBS for cervical cytology, to accelerate adoption and implementation. Additionally, addressing the challenges posed by annotation difficulties in cytology and histology remains critical. Researchers must focus on developing more efficient annotation methods to streamline the process, as this is a significant barrier to clinical adoption. Lastly, bridging the gap between research and clinical practice, particularly in resource-limited settings, will be essential for ensuring equitable access to AI-powered diagnostics. Given the immense potential of AI in these fields, sustained attention and investment are necessary to unlock its full capabilities, revolutionizing cancer and pathology diagnostics and ultimately improving patient care and outcomes.

### 4.6. Limitations

This narrative review follows a structured methodology with well-defined inclusion and exclusion criteria, ensuring a focused and high-quality synthesis of the available literature. While the exclusion of conference proceedings means that some emerging research or preliminary developments may not be captured, this approach prioritizes studies that have undergone rigorous peer review, enhancing the reliability of the findings. Additionally, by focusing on internationally published literature in English, the review ensures broad applicability and comparability across different healthcare settings. However, this may result in the omission of region-specific insights or localized best practices, highlighting an opportunity for future research to explore diverse clinical approaches and treatment protocols in various cultural and healthcare contexts.

## 5. Final Thought: Key Reflections on Digital Cytopathology: Perspectives on Progress and Unresolved Challenges

The findings of this study invite critical reflection from multiple perspectives:

Comparing Digital Cytopathology and Digital Radiology: A primary comparison emerges between digital cytopathology and digital radiology. Radiology has integrated much faster into healthcare due to the adoption of the DICOM standard [77,78], which provides globally standardized protocols and techniques. In contrast, digital cytopathology still faces considerable challenges, as highlighted by both the overview of reviews and the analysis of primary studies. Many studies continue to focus on comparing glass slides to digital methods, emphasizing that digital workflows have yet to become standard in hospital settings, indicating a slower adoption process for cytopathology.

Whole-Slide Imaging (WSI) and Clinical Integration: Whole-slide imaging (WSI) [79,80], a technology developed specifically for digital pathology, holds significant potential for improving pathology workflows. However, much of the research is still focused on its technical evaluation rather than broader clinical integration. Challenges such as standardization, interoperability, and regulatory compliance remain key obstacles to fully implementing WSI in clinical practice, requiring additional focus on overcoming these barriers to maximize its practical application.

The Catalytic Role of Artificial Intelligence: Artificial intelligence (AI) plays a crucial role in advancing digital cytopathology, offering promising opportunities such as large language models (LLMs) and AI-driven chatbots to integrate digital tools into diagnostic workflows [30]. While AI innovations show potential for enhancing diagnostic capabilities [63,70,71,72,73,74,75,76], important areas for further development have emerged. Key to progress will be refining AI-assisted diagnostic solutions, such as AIATBS for cervical cytology, and addressing annotation challenges in cytology and histology. Efficient annotation methods are essential for overcoming barriers to clinical adoption. At the same time, ethical concerns—such as algorithmic transparency, data privacy, and the risk of over-reliance on AI—must be carefully considered to ensure responsible integration. Bridging the gap between research and clinical practice, particularly in resource-limited settings, will help ensure equitable access to AI-powered diagnostics and improve patient care and outcomes. These concerns underscore the importance of thoughtful and effective integration of AI in clinical environments.

Addressing Unresolved Challenges: Despite significant progress in technological innovations and clinical applications, critical issues such as standardization, regulation, and ethical impact remain largely unexplored in particular in AI integration. These challenges must be addressed in alignment with emerging national and international guidelines. Ethical frameworks from organizations such as the American Society of Cytopathology [37,38], global discussions on “algorethics” [81], and international bodies like the WHO [82], the EU [83], and the FDA [84,85] emphasize the importance of ethical AI use, transparency, and equity. These frameworks provide valuable guidance for the responsible adoption of AI tools in digital cytopathology, ensuring that AI technologies are deployed in ways that improve patient outcomes while maintaining ethical standards. Furthermore, initiatives like the NHS AI Ethics Initiative [86], Canada’s Public Health Agency frameworks [87], and China’s ethical norms [88] highlight the need for ethical oversight, data protection, and human rights considerations in AI integration. These principles are particularly critical for digital cytopathology, where AI tools are increasingly central to diagnostics and decision making.

## 6. Conclusions

This study provides a detailed overview of the digital transformation in cytopathology, offering valuable insights into the trends, challenges, and opportunities shaping the field. At the heart of this transformation is the shift toward digitalization, which encompasses a wide range of tools and technologies, including—but not limited to—AI.

One of the study’s main contributions is the identification of key themes emerging from the reviews. Digitalization is reshaping cytopathology by improving diagnostic accuracy, streamlining workflows, and enabling new ways to analyze and interpret data. Tools such as WSI and cloud-based platforms are central to this shift, allowing for more efficient collaboration and data sharing among professionals.

AI plays an important role within the broader context of digitalization. Approximately half of the reviews analyzed highlight its growing impact, particularly in enhancing diagnostic processes, detecting patterns, and supporting clinical decision making. Emerging technologies like LLMs and AI chatbots show potential to further augment digital workflows, providing real-time assistance and automation. However, these advancements also bring challenges, including ethical considerations, data privacy concerns, and the need to address algorithmic biases.

The study also underscores the opportunities that digitalization presents for the future of cytopathology. By adopting digital tools, laboratories can achieve faster, more accurate, and accessible diagnostics. This transformation has the potential to bridge healthcare gaps, especially in underserved regions, by enabling remote diagnostics and telepathology. Additionally, digital platforms facilitate global collaboration, allowing experts to share knowledge and work together on complex cases.

To fully realize the benefits of digital cytopathology, the study identifies several critical steps. These include developing standardized protocols for the use of digital tools, providing comprehensive training for cytopathologists and technologists, and ensuring the validation of new technologies in diverse clinical settings. Collaboration among pathologists, technology developers, and regulatory bodies is also crucial to address challenges and ensure the safe and effective implementation of digital solutions.

In conclusion, the digitalization of cytopathology represents a transformative step forward, offering significant improvements in diagnostic precision, efficiency, and global accessibility. While AI is an important component of this process, the broader focus remains on integrating digital tools to create a more connected and efficient diagnostic ecosystem. By addressing the challenges and leveraging the opportunities outlined in this study, the field is well-positioned to advance both the quality and equity of healthcare worldwide.

## Figures and Tables

**Table 1:** Themes, focus, and description of the overviewed studies.

Ref	Description	Focus on Digitalization	Theme
[30]	Narrative review on the potential of chatbots and NLP in cytology and cytopathology to improve diagnostics, streamline workflows, and enhance patient engagement. The review discusses the use of chatbots and natural language processing (NLP) in the context of cytopathology, highlighting how these tools can improve diagnostic accuracy, reduce errors, and enhance patient interactions.	Application of chatbots and NLP for data extraction, classification, and patient interaction in diagnostics.	Leveraging AI tools like chatbots and NLP to transform cytopathology, reduce errors, and improve accessibility, particularly in patient interactions and data handling.
[31]	Review of image analysis evolution in pathology, highlighting the transformative role of digital tools and AI in diagnostics. This article tracks the development of digital pathology and machine learning (ML) for image analysis, discussing how these technologies improve the analysis of microscopic features and increase diagnostic accuracy.	Use of digital pathology, ML, and advanced imaging for analyzing microscopic features.	AI and digital pathology enhancing diagnostic accuracy and personalization, enabling more precise and individualized diagnoses through improved image analysis.
[32]	Review of preoperative cytological methods for diagnosing salivary gland lesions, emphasizing sensitivity improvements. This review focuses on how advancements in cytology techniques, particularly with preoperative evaluations, have improved diagnostic sensitivity and helped reduce the need for invasive biopsies in complex salivary gland lesions.	Integration of reporting systems and onsite evaluation to ensure sample adequacy.	Preoperative cytology improves diagnostic accuracy for complex salivary lesions by utilizing advanced reporting systems and onsite evaluation to ensure proper sample quality.
[33]	Review of CAD in urine cytopathology for urothelial carcinoma diagnosis and workflow improvement. The paper explores the use of computer-aided detection (CAD) systems and AI to identify biomarkers in urine cytology, improving diagnostic efficiency and workflow by automating the detection of urothelial carcinoma.	CAD and AI tools for identifying biomarkers and reducing errors in urine cytology.	CAD as a transformative solution for urine cytology, increasing diagnostic efficiency by automating and improving the accuracy of identifying cancerous cells.
[34]	Overview of cytopathology advancements in the computational era, focusing on AI and molecular methods. This article reviews how digital pathology, artificial intelligence (AI), and next-generation sequencing are transforming cytopathology, especially in personalized medicine, by providing predictive insights and improving diagnostic capabilities.	Use of AI and next-gen sequencing for diagnosis and predictive insights.	Digital pathology complements molecular investigations in personalized medicine, enhancing the diagnostic process and offering insights into potential treatment outcomes.
[35]	Role of social media in professional growth, networking, and education in cytopathology. This review explores how social media platforms have become valuable tools for education, networking, and collaboration among cytopathologists globally, providing opportunities for sharing knowledge, research, and professional development.	Use of social media for education, networking, and global collaboration.	Social media as a tool for professional development and knowledge dissemination, fostering global collaboration and continuing education in the field of cytopathology.
[36]	Review of the use of endoscopic ultrasound-guided fine needle aspiration biopsy (EUS FNAB) in diagnosing pancreatic neoplasms in pediatric patients. The article examines the role of digital imaging and archives in the diagnosis of pancreatic neoplasms in pediatric patients using EUS FNAB, emphasizing its safety and effectiveness.	Use of digital archives and imaging for diagnosing pancreatic neoplasms.	EUS FNAB as a safe, effective diagnostic method for pediatric pancreatic neoplasms, with digital imaging playing a crucial role in accurate diagnosis.
[37]	Concept paper reviewing AI applications in digital cytology, focusing on best practices and global trends. This paper provides an overview of the growing use of artificial intelligence in digital cytology, covering best practices for AI implementation and the emerging trends that are shaping the future of cytopathology.	Exploration of AI and digital cytology in whole-slide scanning and laboratory workflow.	AI adoption in cytology to improve diagnostic processes and laboratory efficiency, enhancing workflow through the use of digital slide scanning and AI-driven analysis.
[38]	Concept paper on implementing digital cytology in practice, with recommendations from the American Society of Cytopathology. This paper outlines the process of integrating digital cytology into laboratory settings, offering guidance on implementation strategies and the benefits of digital cytology for improving diagnostic outcomes and operational efficiency.	Implementation of digital cytology and AI in laboratory settings for improved practice.	Digital cytology’s integration into practice for better diagnostic outcomes and workflow efficiency, making laboratories more effective and enhancing diagnostic accuracy.
[39]	Exploration of ChatGPT’s role in enhancing pathology workflows, specifically in cancer diagnosis. This article discusses the potential of ChatGPT and similar AI tools in supporting pathology workflows, particularly in improving the speed and accuracy of cancer diagnoses and assisting pathologists in interpreting complex cases.	Integration of AI tools like ChatGPT to enhance pathology workflows and diagnosis.	AI as an assistant tool for pathology, improving cancer diagnosis and diagnostic efficiency by providing real-time support to pathologists.
[40]	Review of digital cytopathology and immunocytochemistry techniques for diagnosing metastatic breast carcinoma. The article examines the combined use of digital cytopathology and immunocytochemistry in diagnosing metastatic breast carcinoma, showcasing how these tools improve diagnostic accuracy and support the development of personalized treatment strategies.	Use of digital cytopathology and immunocytochemistry for enhanced diagnosis.	Digital cytopathology and molecular testing improve metastatic breast carcinoma diagnosis, enabling more precise and personalized therapeutic approaches.
[41]	Review on the role of cytopathology in immunotherapy, specifically in evaluating PD-L1/PD-1 in lung cancer. The review discusses how digital imaging and advanced algorithms are helping pathologists assess PD-L1/PD-1 expression in lung cancer, thereby enhancing the accuracy of immunotherapy treatment planning.	Digital imaging and advanced algorithms to assist in immunotherapy testing.	Digital cytology aids in interpreting PD-L1 assays for lung cancer immunotherapy applications, improving the accuracy of immunotherapy-related diagnostics.
[42]	The COVID-19 pandemic has impeded cytopathology practices and hindered cancer screening and management. The paper explores how the pandemic disrupted cytopathology practices, caused delays in cancer screening, and led to a shift towards digital platforms for remote diagnosis and education, helping maintain workflow during the crisis.	The pandemic led to a shift toward digital platforms for education and diagnostic purposes, along with delays in cancer diagnoses and screenings.	Impact of COVID-19 on cytopathology practices, highlighting the importance of digital tools in maintaining workflow during crises and mitigating diagnostic delays.
[43]	Current status of machine learning in thyroid cytopathology. The review assesses the current state of machine learning (ML) applications in thyroid cytology, focusing on fine needle aspiration biopsy (FNAB) specimens, and highlights promising results in improving diagnostic accuracy and workflow efficiency.	Evaluation of machine learning algorithms for thyroid cytology, particularly fine needle aspiration biopsy.	ML algorithms improve diagnostic accuracy and workflow efficiency in thyroid cytopathology, particularly in cancer diagnosis and classification.
[44]	Artificial intelligence-based tools applied to pathological diagnosis of microbiological diseases. The paper reviews AI applications in microbiological pathology, discussing how AI tools help pathologists identify microorganisms from cytological specimens, particularly in resource-limited settings where manual identification is challenging.	AI applications in identifying microorganisms from cytological specimens, especially in resource-limited settings.	AI-based tools enhance the identification of microbiological diseases in cytopathology, particularly in regions with limited resources, enabling faster and more accurate diagnoses.
[45]	Digital diagnostic cytopathology: has the pandemic brought us closer? This article examines how the COVID-19 pandemic accelerated the adoption of digital cytopathology, especially for educational purposes and remote diagnostic reporting, and the long-term benefits of these changes.	Increased adoption of digital cytopathology during the pandemic, particularly in academic and educational settings.	The pandemic accelerated digital cytopathology adoption, enhancing educational and diagnostic capabilities and offering long-term benefits for the field.
[12]	Digital cytology: current status and future prospects. This paper reviews the current status of digital cytology, including virtual microscopy and whole-slide imaging (WSI), and discusses its potential future impact on diagnosis, pathology training, and research.	Growing role of digital cytology in diagnostic workflows, with an emphasis on whole-slide imaging.	Digital cytology offers improvements in diagnosis, training, and pathology education, with whole-slide imaging emerging as a key technology for future cytology practice.
[46]	Relevance of the College of American Pathologists’ guidelines for validating whole-slide imaging for diagnostic purposes to cytopathology. The article reviews the College of American Pathologists’ (CAP) guidelines for validating whole-slide imaging (WSI) systems in cytopathology, emphasizing the need for further research and validation studies.	Review of CAP guidelines for whole-slide imaging (WSI) validation in cytopathology, with an emphasis on further research.	CAP guidelines guide WSI adoption in cytopathology, emphasizing the need for additional validation studies to ensure the technology’s reliability for diagnostic purposes.
[47]	This systematic review examines the role of AI and whole-slide imaging (WSI) in enhancing diagnostic accuracy for thyroid cytopathology, particularly in cases of atypia of undetermined significance/follicular lesions of undetermined significance (AUS/FLUS).	The study investigates the integration of AI and WSI to improve the diagnostic accuracy of thyroid cytopathology, focusing on indeterminate thyroid lesions.	AI and WSI integration shows promise in enhancing diagnostic accuracy and reducing uncertainty in thyroid cytology, with a focus on improving precision in AUS/FLUS cases.
[48]	This short review discusses the use of digital cytopathology through Z-stack scanning and AI, aimed at improving the imaging and detection of cancerous cells in cytology specimens.	The study explores the application of Z-stack scanning, with or without extended focusing, paired with AI technology to improve cancer detection and molecular analysis in cytology specimens.	The integration of Z-stack scanning and AI for more precise detection and molecular analysis in digital cytology, with an emphasis on improving cancer diagnostics.
[49]	This review outlines the unique challenges of applying AI to digital cytology, including data handling, large file sizes, ethical issues, and the need for regulatory frameworks.	The article addresses the technical and ethical challenges involved in incorporating AI into digital cytology, such as large image file sizes, AI model validation, and data security concerns.	The review highlights the specific challenges and ethical considerations in applying AI to digital cytology, emphasizing the need for robust data handling and regulatory frameworks.

**Table 3:** Categorization of emerging opportunities.

Thematic Category of Opportunities	Key Opportunities	Potential Impact	Ref.
AI and Machine Learning Integration in Diagnostic Accuracy	AI-powered technologies (NLP, chatbots) enhance patient engagement and streamline workflows by automating medical data extraction.	Reduces human error, improves patient communication, and optimizes clinical decision making.	[30]
AI and Machine Learning Integration in Diagnostic Accuracy	Machine learning improves diagnostic accuracy, prognostic predictions, and personalized treatment strategies.	Facilitates complex tissue evaluation and enhances precision medicine.	[31]
AI and Machine Learning Integration in Diagnostic Accuracy	Greater AI adoption in cytology workflows enhances diagnostic accuracy and supports pathologists in decision making.	Increases lab efficiency and reduces diagnostic variability.	[37]
AI and Machine Learning Integration in Diagnostic Accuracy	AI-driven cancer pathology diagnostics aid pathologists through advanced algorithms and digital slide integration.	Speeds up cancer diagnosis and enhances accuracy.	[39]
AI and Machine Learning Integration in Diagnostic Accuracy	Machine learning improves thyroid cytology classification and enhances workflow efficiency.	Reduces subjectivity in diagnoses and improves thyroid cancer detection.	[43]
AI and Machine Learning Integration in Diagnostic Accuracy	AI assists in microorganism detection, particularly in resource-limited settings.	Enables faster and more accurate infectious disease diagnosis.	[44]
AI and Machine Learning Integration in Diagnostic Accuracy	AI integration in thyroid cytopathology reduces errors and enhances differentiation of indeterminate lesions.	Increases precision and consistency in diagnostic outcomes.	[47]
AI and Machine Learning Integration in Diagnostic Accuracy	Digital pathology and AI streamline cytology practice, improving workflow and decision making.	Boosts efficiency and reduces diagnostic errors.	[49]
Digital Pathology and Technology in Molecular Diagnostics	Digital cytopathology complements molecular investigations and enhances diagnostic precision.	Supports personalized medicine and treatment optimization.	[34]
Digital Pathology and Technology in Molecular Diagnostics	Immunocytochemistry and digital pathology improve breast carcinoma diagnosis and treatment approaches.	Increases diagnostic precision and personalized therapeutic decisions.	[40]
Digital Pathology and Technology in Molecular Diagnostics	Digital cytopathology enhances immune marker evaluation for lung cancer, supporting PD-L1 testing.	Improves immune profiling and targeted therapies.	[41]
Digital Pathology and Technology in Molecular Diagnostics	AI and digital pathology facilitate cancer cell detection and targeted gene analysis.	Enables future automation of molecular diagnostics.	[48]
Improved Diagnostic Workflow and Efficiency	Advanced onsite cytology evaluation improves diagnostic accuracy for salivary gland lesions.	Enhances early detection and risk stratification.	[32]
Improved Diagnostic Workflow and Efficiency	Computer-assisted diagnosis (CAD) systems improve diagnostic accuracy in urothelial carcinomas.	Streamlines workflows and enhances patient outcomes.	[33]
Improved Diagnostic Workflow and Efficiency	Digital cytology enhances workflow efficiency through whole-slide imaging and global accessibility.	Facilitates remote diagnosis and collaborative cytopathology.	[38]
Improved Diagnostic Workflow and Efficiency	Whole-slide imaging (WSI) improves training for pathology professionals.	Supports education and enhances diagnostic consistency.	[12]
Professional Development and Collaboration	Social media facilitates global networking, academic visibility, and knowledge dissemination.	Fosters real-time collaboration and continuous education.	[35]
Professional Development and Collaboration	Remote and digital platforms enhance cytopathology education and lab protocols.	Expands accessibility and adaptability in training.	[42]
Professional Development and Collaboration	CAP guidelines standardize whole-slide imaging (WSI) validation for clinical practice.	Improves diagnostic accuracy and consistency in digital pathology.	[46]
Advancements in Pediatric and Rare Disease Diagnostics	Endoscopic ultrasound-guided fine-needle aspiration (EUS FNAB) improves pediatric pancreatic neoplasm diagnosis.	Offers a minimally invasive, high-precision diagnostic approach.	[36]
Impact of the COVID-19 Pandemic	The pandemic accelerated digital cytopathology adoption for education and remote diagnostics.	Increased flexibility, efficiency, and regulatory adaptations.	[45]
